# Multicentric ^68^Ga-PSMA PET radiomics for treatment response assessment of ^177^Lu-PSMA-617 radioligand therapy in patients with metastatic castration-resistant prostate cancer

**DOI:** 10.3389/fnume.2023.1234853

**Published:** 2023-09-14

**Authors:** Robin Gutsche, Gizem Gülmüs, Felix M. Mottaghy, Florian Gärtner, Markus Essler, Dirk von Mallek, Hojjat Ahmadzadehfar, Philipp Lohmann, Alexander Heinzel

**Affiliations:** ^1^ Institute of Neuroscience and Medicine (INM-4), Forschungszentrum Juelich, Juelich, Germany; ^2^ RWTH Aachen University, Aachen, Germany; ^3^ Department of Nuclear Medicine, University Hospital RWTH Aachen, Aachen, Germany; ^4^ Department of Nuclear Medicine, University Hospital Bonn, Bonn, Germany; ^5^ Department of Nuclear Medicine, Klinikum Westphalen, Dortmund, Germany; ^6^ Department of Nuclear Medicine, University Hospital Halle (Saale), Halle (Saale), Germany

**Keywords:** PET/CT, artificial intelligence, machine learning, metastases, prognosis

## Abstract

**Objective:**

The treatment with ^177^Lutetium PSMA (^177^Lu-PSMA) in patients with metastatic castration-resistant prostate cancer (mCRPC) has recently been approved by the FDA and EMA. Since treatment success is highly variable between patients, the prediction of treatment response and identification of short- and long-term survivors after treatment could help tailor mCRPC diagnosis and treatment accordingly. The aim of this study is to investigate the value of radiomic parameters extracted from pretreatment ^68^Ga-PSMA PET images for the prediction of treatment response.

**Methods:**

A total of 45 mCRPC patients treated with ^177^Lu-PSMA-617 from two university hospital centers were retrospectively reviewed for this study. Radiomic features were extracted from the volumetric segmentations of metastases in the bone. A random forest model was trained and validated to predict treatment response based on age and conventionally used PET parameters, radiomic features and combinations thereof. Further, overall survival was predicted by using the identified radiomic signature and compared to a Cox regression model based on age and PET parameters.

**Results:**

The machine learning model based on a combined radiomic signature of three features and patient age achieved an AUC of 0.82 in 5-fold cross-validation and outperformed models based on age and PET parameters or radiomic features (AUC, 0.75 and 0.76, respectively). A Cox regression model based on this radiomic signature showed the best performance to predict overall survival (C-index, 0.67).

**Conclusion:**

Our results demonstrate that a machine learning model to predict response to ^177^Lu-PSMA treatment based on a combination of radiomics and patient age outperforms a model based on age and PET parameters. Moreover, the identified radiomic signature based on pretreatment ^68^Ga-PSMA PET images might be able to identify patients with an improved outcome and serve as a supportive tool in clinical decision making.

## 1. Introduction

Prostate cancer affects millions of men worldwide and is the second-common malignant tumor after lung cancer.

A promising approach to the treatment and diagnosis of prostate cancer at the castration-resistant stage specifically targets the type II gylcoprotein prostate-specific membrane antigen (PSMA). It is expressed at a low level in normal prostatic tissues and nonprostatic tissues, with a 100–1,000-fold increased expression in prostate cancer tissues (1, 2). Thus, PSMA represents a theranostic target for imaging diagnostics and targeted radionuclide therapy. Recently, the US Food and Drug Administration (FDA) as well as the European Medicines Agency (EMA) approved the first PSMA-targeted radiopharmaceutical based on the results of the phase III VISION trial for treatment of patients with PSMA-positive metastatic castration-resistant prostate cancer (mCRPC) (3).

Besides the effective treatment, another crucial aspect of the theranostic approach is a personalized treatment based on the molecular properties of tumors in an individual patient. ^68^Ga-labelled PSMA positron emission tomography/computed tomography (PSMA PET/CT) has been applied successfully for primary staging, image-guided radiotherapy, and surgery in recurrent prostate cancer and advanced-stage metastatic prostate cancer (4–6). Thus, there is growing evidence of integrating PSMA PET/CT imaging into personalized prostate cancer treatment concepts (7). Moreover, it is recommended for patient selection and treatment monitoring by the European Association of Nuclear Medicine (EANM) procedure guidelines for radionuclide therapy with ^177^Lu-labelled PSMA-ligands (^177^Lu-PSMA) (8).

So far, PSMA PET studies addressing patient selection, prognosis, or treatment response for ^177^Lu-PSMA predominately focused on the use of SUV-based analyses related to PET Response Criteria in Solid Tumors (PERCIST) as well as total tumor volumes (TTV) (9–12).

Radiomics is a subdiscipline within the broad field of artificial intelligence, and it has also demonstrated its potential in nuclear medicine and oncology. It permits assessing tumor heterogeneity quantitatively by extracting a large number of image features from imaging data using various mathematical algorithms. Based on these quantitative features, machine learning models can be generated that may allow the prediction of outcome parameters such as treatment response or survival (13, 14).

So far, studies applying radiomic analyses to PSMA PET data in prostate cancer have demonstrated potential applications for detection, risk assessment, and prognosis at initial diagnosis (15–17). Only a few studies have evaluated the potential of PET radiomics for patient selection for ^177^Lu-PSMA treatment (18–20). Thereof, only one study predicted the treatment response in a small number of subjects (*n* = 21) (20), and another one evaluated the prognostic potential of survival models based on radiomic features (19).

The aim of this study was to compare age and common PET parameters for response prediction to ^177^Lu-PSMA treatment with radiomic features. Secondly, the study aimed to assess the prognostic value of the radiomic signature for the differentiation between responders and non-responders and evaluate its contribution to an optimized patient selection.

## 2. Materials and methods

### 2.1. Patients

This study was approved by the institutional review board of the University Hospital Aachen. Given the type of study (a retrospective analysis), the need for written informed consent was waived. All procedures were performed in accordance with the ethical standards of the institutional and/or national research committees and with the principles of the 1964 Declaration of Helsinki and its later amendments or comparable ethical standards.

Patients were retrospectively recruited from the Departments of Nuclear Medicine at the University Hospitals of Bonn and Aachen who received ^68^Ga-PSMA PET/CT from November 2014 through January 2018, followed by a treatment of ^177^Lu-PSMA-617 consisting of three or four cycles.

Criteria for selecting the patients were as follows: We included mCRPC patients with bone metastases for whom imaging using ^68^Ga-PSMA PET/CT was performed before the first cycle of ^177^Lu-PSMA-617 treatment. Only patients were considered who had been treated with at least one of the new-generation antihormonal drugs (abiraterone or enzalutamide) before the ^177^Lu-PSMA-617 treatment. In addition, eligibility criteria required PSA measurements before and after the third or fourth treatment cycle. The timespan between the ^68^Ga-PSMA PET/CT and the PSA measurement had to be four weeks or less. All patients had disease progression despite first- or second-line chemotherapy (docetaxel or cabazitaxel), or the patients were ineligible for chemotherapy or ^223^Ra-dichloride. Patients with tumor lesions in bone metastasis with an SUVmax < 3 and a lesions size <1 ml were excluded. All patient characteristics are shown in Table 1.

**Table 1:** Patient characteristics.

Characteristic	Training-set
Number of patients	45
Age (median, range) (years)	72 (51–87)
OS (median, range) (months)	16 (5–37)
Median follow-up (months)	16
Censored	12 (27%)
MTV (median, range) (ml)	32 (1.5–52)

MTV, metabolic tumor volume; OS, overall survival.

### 2.2. ^177^Lu-PSMA-617 treatment

The treatment and imaging procedures have been described previously (10). Briefly, the ^177^Lu-PSMA-617 treatment was performed according to the German consensus guidelines in both centers (21). The detailed treatment protocol is described in (21).

### 2.3. ^68^Ga-PSMA PET/CT imaging

The ^68^Ga-PSMA-HBED-CC tracer for the PET/CT scans was produced by the in-house radiopharmacy (22). Patients received an intravenous injection of approximately 2 MBq/kg body weight of 68Ga-PSMA 45 minutes before the start of the PET/CT scan. Patients were scanned in caudocranial orientation with raised arms. Attenuation correction was performed using the CT data. Image data at both centers were acquired according to international standard guidelines applying EARL for harmonization across centers.

### 2.4. MTV segmentation

We defined any focal bone uptake of ^68^Ga-PSMA with an SUVmax ≥3 as bone metastases.

An experienced nuclear medicine physician (AH, board-certified with >10 years of experience in PET/CT) identified and segmented the bone lesion with the highest SUVmax for each scan using HERMES HYBRID VIEWER PDR 5.1.0 Hermes Medical Solutions Inc., Greenville, United States). The segmentation was performed using PMOD 3.13 (PMOD Technologies LLC, Zurich, Switzerland) by creating a VOI around the bone lesion with the highest SUVmax that contained all voxels of this lesion with a SUVmax > 3. SUVmax and SUVmean of the identified bone lesions were extracted.

### 2.5. Radiomic feature extraction

Feature extraction was performed with the open-source Python package pyradiomics (version 3.0.1) (23). No spatial resampling of the PET images was performed. Absolute-intensity discretization was performed using a bin width of 0.15. On the original image, 107 features were calculated for each volume of interest (VOI), including 18 first-order statistics, 14 shape features, 24 features from the grey level co-occurrence matrix (GLCM), 16 features from the grey level run length matrix (GLRLM), 16 features from the grey level size-zone matrix (GLSZM), 5 features from the neighborhood grey level different matrix (NGLDM), and 14 features from the grey level dependence matrix (GLDM). Furthermore, high-pass filters using the Laplacian-of-Gaussian image filter (LoG; sigma, 1–5), as well as the discrete 3-dimensional wavelet transformation with the “coifl” wavelet and reconstruction of the higher spatial frequency content in all directions resulting in 8 different wavelet decompositions, were applied, and all features (except the shape features) were also calculated on the filtered images. In total, 1,316 features were calculated for each VOI: 107 features on the original image, 465 features on the LoG-filtered image, and 744 features on the wavelet-transformed images (93 features on each of the 8 different wavelet decompositions).

### 2.6. Feature selection

To avoid the usage of non-reproducible radiomic features, we followed the process of a test-retest analysis described previously (24). In short, data augmentation methods were used to generate a modified version of the original image. Extracted features from the original and modified versions of the image were then analyzed for repeatability. Features were considered repeatable if the lower and upper limits of the intraclass correlation coefficient (95% confidence interval) were in the range of 0.91 and 1.00. The feature correlation was assessed by the Pearson correlation coefficient. From 1,302 extracted features, 364 repeatable features were identified. Further, features were considered uncorrelated if the Pearson correlation coefficient was below 0.9. Of the 364 repeatable features, 330 showed a high linear correlation. Finally, the features with the highest mutual information for predicting responses were selected, resulting in 34 features that were included in the modeling process.

### 2.7. Response prediction

With regard to PSA, we defined the treatment response according to the Prostate Cancer Work Group 3 criteria, i.e., a PSA decline of ≥50% compared to the baseline was considered a response (25). Prior to training the random forest model, all radiomic features were standardized by subtracting the mean and dividing the standard deviation of the training data. A five-fold stratified shuffled cross-validation was performed, with subsequent feature selection based on the validation fold showing the best performance. This process was repeated until the average validation metric did not improve further, and the model with the best performing features and hyperparameters (*n*_estimators = 500, max_depth = 5, min_samples_split = 3) was retrained on the complete training data set. The random forest model used a radiomic signature of three features (first-order range; first-order mean absolute deviation; GLCM inverse variance).

The prediction of treatment response was derived from three individual models: a model based on a combination of patient age and the PET parameters SUVmean and SUVmax; a radiomic model based on the previously described radiomic signature; and a model based on a combination of the radiomic signature and the patient age. The performance of the models was evaluated by comparing the area under the curve of the receiver operating characteristics (AUC). All processing steps were implemented in Python (scikit-learn, version 0.24.1). Further details are provided in (24).

### 2.8. Survival prediction

Patient survival time was determined starting from the pre-therapeutic PET/CT until patient death or until the last patient contact if the patient was still alive (censored). The complete dataset was randomly split into a training set and a test set (60/40). Prognosis models were calculated, and statistical analysis was performed using the Python library scikit-survival (version 0.15.0) (26). Multiple Cox's regression hazard models were trained from age and PET parameters, the previously identified features of the classification task (response vs. no response), or a combination on the training set. Finally, hazard ratios were predicted on the test set. Performance was evaluated based on the concordance index (c-index). For the sake of illustration, the risk scores were dichotomized by their median, and the Kaplan-Meier curves of the resulting low- and high-risk groups were computed for the training and test sets. A log-rank test was used to evaluate statistical differences between low- and high-risk groups.

## 3. Results

### 3.1. Patient characteristics

**Figure 1:**
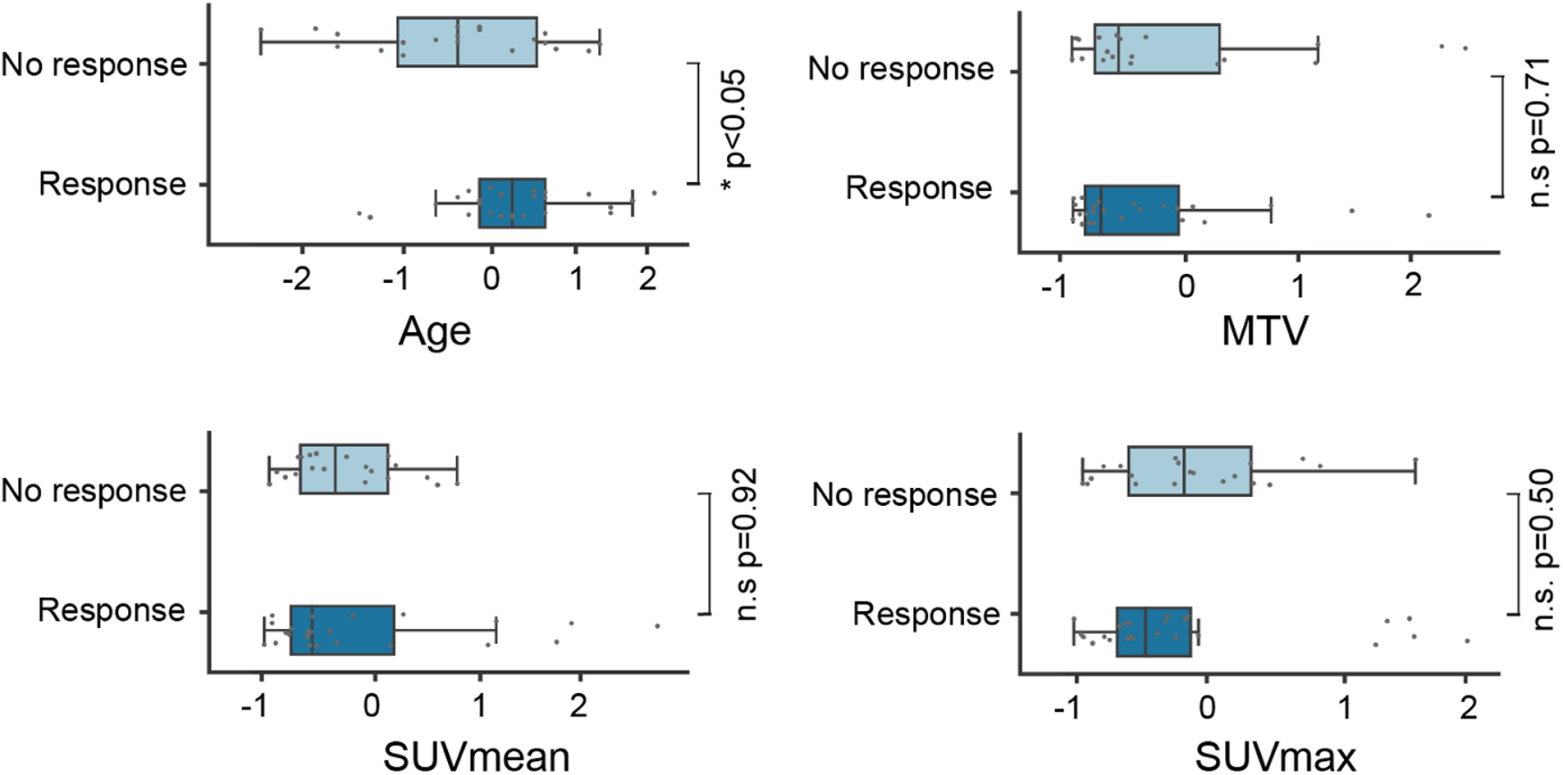
Feature distribution for age and PET parameters. SUV, standardized uptake value; MTV, metabolic tumor volume.

We initially identified 80 patients for potential inclusion in this study. In 35 patients, PSA measurements were taken later than four weeks after the PET/CT, and had SUVmax values <3 or small lesions <1 ml. In total, 45 patients were subjected to further analyses and received an average dose of 5.8 GBq of ^177^Lu-PSMA-617 (range: 4.0–7.3 GBq). Among these patients, 60% had received previous treatments with docetaxel, 24% with cabazitaxel, 67% with abiraterone, 71% with enzalutamide, 42% with both abiraterone and enzalutamide, 42% with ^223^Radium, and 49% with external radiation therapy. A total of 25 patients showed a response to the ^177^Lu-PSMA-617 treatment (55%). Responding patients had a median metabolic tumor volume (MTV) of 23.3 ml (IQR25–75, 11.1–77.3 ml) in contrast to 33.3 ml (IQR25–75, 17.1–102.5 ml) for non-responding patients (*p* = 0.71) (Figure 1). The mean SUVmean ± SD and SUVmax ± SD were 9.2 ± 5.7 and 32.9 ± 32.0 for responding patients and 7.8 ± 2.4 and 29.0 ± 15.7 for non-responding patients (Figure 1). No statistically significant differences were detected between both groups for SUVmean and SUVmax (*p* = 0.92; *p* = 0.50). Patients showing a response were significantly older than non-responding patients (mean age ± SD, 74.2 ± 6.5 vs. 68.7 ± 8.7; *p* < 0.05) (Figure 1).

### 3.2. Response prediction

**Figure 2:**
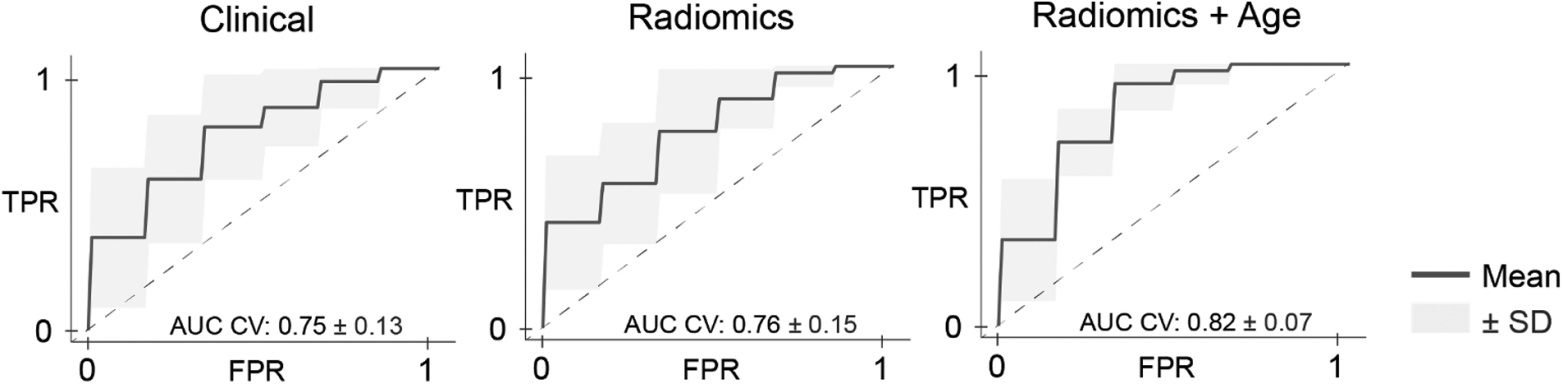
Receiver operating characteristic curves for response prediction models. AUC, area under the receiver operating characteristic curve; CV, cross-validation; FPR, false positive rate; SD, standard deviation; TPR, true positive rate.

**Figure 3:**
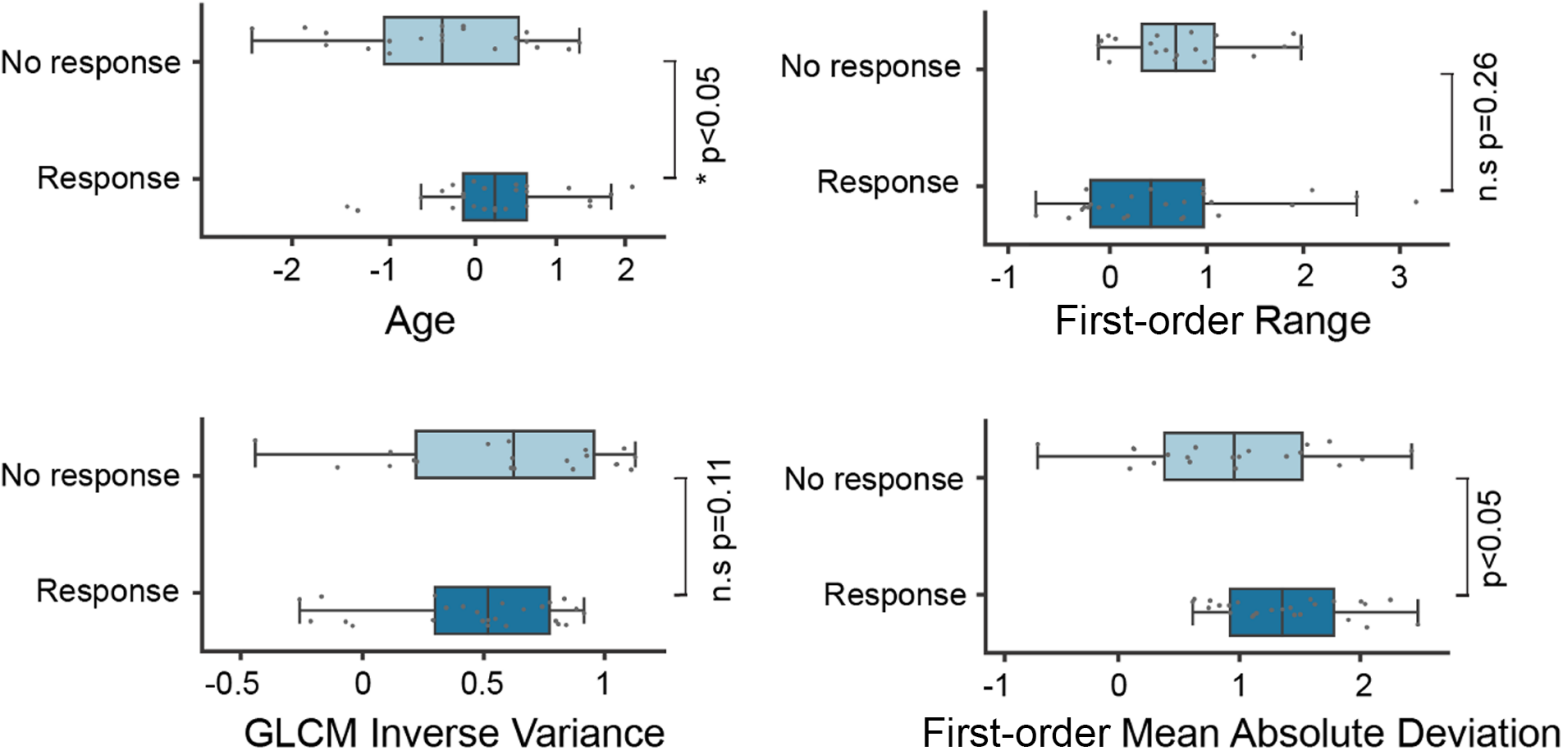
Distribution of the radiomic features and patient's age between responders and non-responders. GLCM, gray-level co-occurrence matrix.

Univariate feature analysis of patient age, SUVmean, and SUVmax revealed AUCs of 0.63, 0.51, and 0.56, respectively (Table 2). A random forest model based on a combination of all three features resulted in a mean AUC ± SD of 0.75 ± 0.13 during 5-fold cross-validation (Table 3 and Figure 2). Regarding this model, we compared a random forest model based on a radiomic signature of three features in its ability to predict response to ^177^Lu-PSMA-617 treatment on pretreatment ^68^Ga-PSMA PET scans. The radiomic model achieved a similar performance as the previous model, with an AUC ± SD of 0.76 ± 0.15 (Table 3 and Figure 2). A random forest model based on a combination of the radiomic signature and the clinical feature patient age resulted in an AUC ± SD of 0.82 ± 0.07 (Table 3 and Figure 2). The feature distribution of the radiomic signature is shown in Figure 3.

**Table 2:** Results of univariate response prediction based on age and PET parameters.

Feature	AUC	Threshold	ACC	SEN	SPE
Age	0.63	69	0.71	0.84	0.55
SUVmean	0.51	7.8	0.58	0.64	0.50
SUVmax	0.56	25.1	0.62	0.60	0.65

ACC, accuracy; AUC, area under the receiver operating characteristic curve; SEN, sensitivity; SPE, specificity; SUV, standardized uptake value.

**Table 3:** Cross-validation results of response prediction for individual models.

CV-fold	Age + PET parameters (*n* = 3)				Radiomics (*n* = 3)				Radiomics + age (*n* = 4)			
	AUC	ACC	SEN	SPE	AUC	ACC	SEN	SPE	AUC	ACC	SEN	SPE
1	0.85	86	100	67	0.93	86	75	100	0.88	86	100	67
2	0.92	86	75	100	0.90	86	100	67	0.92	86	88	83
3	0.73	79	75	83	0.67	64	63	67	0.77	86	100	67
4	0.60	71	88	50	0.63	71	75	67	0.75	79	88	67
5	0.65	71	50	100	0.67	71	88	50	0.79	79	75	83
Mean	**0** **.** **75**	79	78	80	**0** **.** **76**	76	80	70	**0** **.** **82**	83	90	73
±SD	0.13	7	19	22	0.15	10	14	18	0.07	4	10	9

Bold values represent the mean values of the cross-validation folds. CV, cross-validation; AUC, area under the receiver operating characteristic curve; SEN, sensitivity; SPE, specificity.

### 3.3. Survival prediction

**Figure 4:**
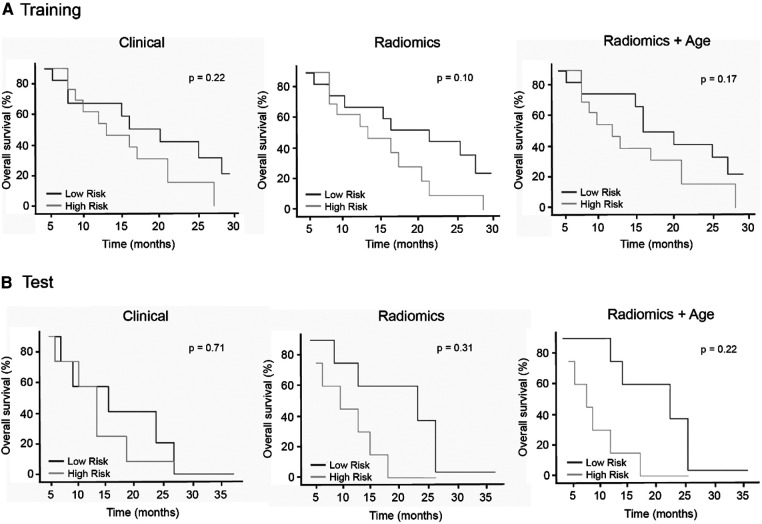
Kaplan-Meier survival curves for risk prediction based on multiple Cox regression models for the training data (**A**, top row) and the test data (**B**, bottom row).

**Figure 5:**
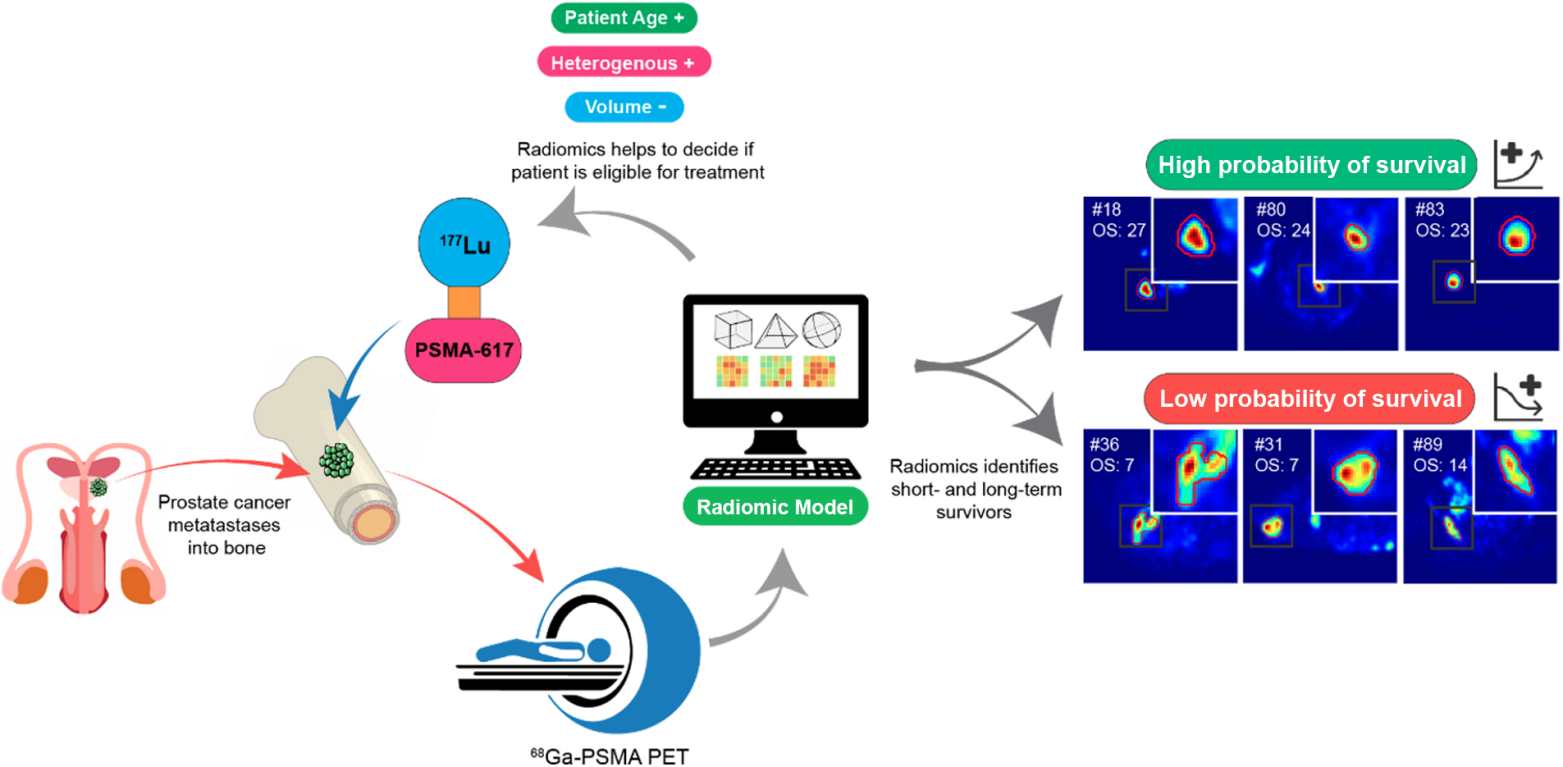
Potential clinical implementation of the workflow combining the response assessment and prognosis estimation, as well as representative PET examples for lesions identified as high survival and low survival probability.

To test if the identified radiomic signature also has a prognostic value, we calculated three individual multiple Cox regression models based on age and PET parameters: the radiomic signature and a combination of the radiomic signature and the patient age. The survival model based on a combination of radiomic signature and patient's age outperformed the models based on age and PET parameters or radiomic features alone, resulting in a concordance index of 0.64 for the combined model, 0.56 for the model based on age and PET parameters, 0.62 for the radiomic model in the training set, 0.67 for the combined model, 0.50 for the model based on age and PET parameters, and 0.65 for the radiomic model in the test set (Table 4). None of the Kaplan–Meier curves divided into low- and high-risk groups based on the risk scores estimated by the models showed significant differences between the groups (Figure 4). Representative lesions for low- and high-risk patients and a potential clinical workflow combining the response assessment and estimation of prognosis are provided in Figure 5.

**Table 4:** Results of prognosis prediction for multiple Cox regression models based on age, PET parameters, radiomics.

	Age + PET parameters (*n* = 3)	Radiomics (*n* = 3)	Radiomics + age (*n* = 4)
C-index training	0.56	0.62	0.64
Log-rank *p*-value	0.22	0.10	0.17
C-index test	0.50	0.65	0.67
Log-rank *p*-value	0.71	0.31	0.22

## 4. Discussion

Our study showed that a machine learning model based on a combination of radiomic features extracted from pretreatment PSMA PET and the patient age may predict the treatment response to ^177^Lu-PSMA therapy. In addition, the results indicate a trend for possible prediction of long vs. short survival time after treatment.

Studies on treatment with ^177^Lu-PSMA have shown impressive efficacy. Therefore, it is considered a “beacon of hope” even in intensely pre-treated patients (27). However, it is known that a significant percentage of treatments will not be successful (28). Moreover, especially intensely pre-treated patients are vulnerable to possible severe side effects. Thus, careful patient selection regarding the beneficial treatment outcome is of the utmost importance. Currently, patient selection, among other clinical parameters, is based on tracer accumulation in tumor sites compared to physiological accumulation in normal liver tissue in the pretreatment PSMA PET (8, 28). Additionally, studies investigated the use of SUV-based parameters. However, so far, the results are contradictory. A recent study found a relationship between pretherapeutic accumulation and imaging-based responses on PSMA PET for primary tumors, lymph nodes, bones and visceral metastases (29). This is in line with other recent findings (11, 30). In contrast, former studies did not find significant image-derived predictive factors (31, 32).

Regarding the prediction of the ^177^Lu-PSMA-617 treatment response, our results indicate that the identified radiomic features, first-order range and mean absolute deviation, as well as the GLCM inverse variance, are superior to conventional SUV-based parameters such as SUVmax and SUVmean. In our analysis, the prediction model based on radiomic features, especially in combination with the clinical parameter age, showed the highest diagnostic accuracy.

Our results are in line with other studies indicating the role of radiomics in PSMA PET-based pretreatment patient selection. Khurshid et al. (18) found that textural heterogeneity parameters extracted from metastatic bone lesions in mCRPC patients correlated with the change in PSA levels following therapy. Their results indicate that the more heterogeneous the tumor with regard to its PSMA expression, the more responsive it is to ^177^Lu-PSMA-617 therapy. These results agree with the findings of our study, where more responsive tumors seem to have more heterogeneous PSMA uptake. This assumption is supported by a wider first-order range and lower values for the texture feature GLCM inverse variance, a description of local homogeneity. Similar results were also found by Roll et al. (20).

In a follow up study of the same group, Mozamei et al. (19) extracted radiomic features in a patient group with histologically confirmed advanced prostate carcinoma from all hotspots, including the primary tumor as well as the metastatic lesions in different organs. They identified a multiple Cox regression model based on SUVmin and first-order kurtosis, which showed similar prognostic values compared to other clinical features such as Hb1, CRP1, ECOG1, and SUVmean. Neither first-order kurtosis nor SUVmin showed a prognostic value in our patient group. However, due to differences in methodology in various aspects, such as VOI-definition, statistics, reference standard, etc., a direct comparison between the results is difficult.

According to our analysis, SUV-based parameters are outperformed by radiomic features, especially in combination with the clinical parameter age. This underlines the importance of integrating clinical parameters into machine learning models to potentially increase predictive power. Promising clinical parameters that could be of interest for future studies might be blood-based parameters such as hemoglobin or the ECOG performance scale (32). Moreover, tumor-specific parameters such as the Gleason score, which reflects tumor aggressiveness, may also be considered (33). However, Rolls et al. did not find a prognostic impact of the Gleason score, patients' performance status, or hemoglobin (20). The authors argue that this might be partly due to their specific sample containing mostly patients with a very high Gleason score in the majority of patients (median, nine) with a mostly high performance status (median, one). This underlines the importance of analyzing data from heterogeneous patient samples in large multicenter studies. Our study contains data from two different institutions, including different PET/CT scanners. This may partly reduce a possible selection bias and permit a more heterogeneous patient sample. However, this may also lead to an increase in variance in the data, thereby requiring larger sample sizes. This could explain why we did not find significant results for the prediction of overall survival in contrast to other groups with more homogeneous patient samples (19, 20).

Studies have demonstrated that higher pretreatment PSMA-PET uptake is associated with improved overall survival, thereby establishing a cut-off of SUVmean >10 for optimized patient selection (34). Yet, despite optimal standard PET/CT imaging, some patients demonstrate primary disease progression (35). Moreover, some of the patients with non-optimal standard PET/CT imaging might still benefit from treatment (36). Therefore, we aimed to identify additional factors based on the radiomic features that are independent of the established SUVmean threshold.

In addition to survival, treatment-related quality of life represents another important outcome parameter that we did not address due to a lack of data. As quality of life is of particular interest, especially regarding a palliative treatment approach, future studies should address this issue.

Some limitations of the study must be noted. The retrospective design entails several methodological disadvantages, including a potential selection bias. The observation that responders were older than non-responders in this study is of interest, but the reasons for this remain unclear. The number of participants in the study is not sufficient to further investigate whether treatment response is correlated with age or not. Only prospective studies in a multi-center setting could ultimately solve this issue. Moreover, the segmentation method of the tumoral lesions was based on PET using a fixed threshold permitting reproducibility. However, due to the possible inter-individual variety in tumor biology, an individualized patient-based threshold might be more adequate in some patients. Furthermore, although performing a multistep feature dimension reduction, cross-validation in the training phase, and the use of a separate test data set for survival prediction, the number of patients remains relatively small. Thus, the generalizability of the statistical model, especially the radiomic signature, needs to be considered in studies with larger sample sizes, including datasets from other external institutions.

## 5. Conclusion

The developed radiomic model using data from pretreatment PSMA PET might be of value for response prediction and patient selection for Lu-PSMA therapy. Although further validation of the results is warranted, due to its potential for automated and objective image evaluation, its integration into the theranostic workflow for patient evaluation should be considered. However, prospective studies with larger sample sizes are needed prior to a potential translation to clinical routine, which should also attempt to correlate the identified radiomic features with histologic specimens.

## Data Availability

The datasets for this article are not publicly available due to concerns regarding participant/patient anonymity. Requests to access the datasets should be directed to the corresponding author.
